# Gastrointestinal stromal tumor of the duodenum presenting with shock and massive upper and lower gastrointestinal bleeding: a case report and review of the literature

**DOI:** 10.1186/s13256-024-04597-x

**Published:** 2024-06-22

**Authors:** Yasser Abou Elsoud Mohamed, Muhammad Mostafa Abdelghaffar, Samar S. Khalaf, Ahmed F. Amin, Mostafa Adel Mostafa, Ola Harb, Asmaa Hussein Mohamed, Ahmed Raafat Abdelfattah

**Affiliations:** 1 Consultant of Gastroenterology and Endoscopy at Mahala Hepatology Teaching Center, Gharbiya, Egypt; 2 Consultant of Gastroenterology and Endoscopy at Ahmed Maher Teaching Hospital, Cairo, Egypt; 3 Department of Biochemistry, Faculty of Pharmacy, Heliopolis University, Cairo, Egypt; 4 Department of Anesthesia and ICU, Faculty of Medicine, Zagazig University, Zagazig, Egypt; 5 Department of Pathology, Faculty of Medicine, Zagazig Universit, Zagazig, Egypt; 6 Department of General Surgery, Faculty of Medicine, Zagazig University, Zagazig, Egypt

**Keywords:** Duodenal GIST, Bleeding, Endoscopy

## Abstract

**Background:**

Due to rarity of duodenal GISTs, clinicians have few information about its clinical features, diagnosis, management and prognosis.

**Case report:**

We report a case of promptly diagnosed duodenal GIST in a 61-year-old Egyptian man presented shocked with severe attack of hematemesis and melena. Upper gastroduodenal endoscopy was done and revealed a large ulcerating bleeding mass at first part of duodenum 4 hemo-clips were applied with good hemostasis.

An exploratory laparotomy and distal gastrectomy, duodenectomy and gastrojejunostomy were performed. The morphology of the mass combined with immunohistochemistry was consistent with duodenal gastrointestinal stromal tumours (GISTs) of high risk type. The patient is on amatinib one tablet daily and he was well with no evidence of tumor recurrence.

**Conclusion:**

despite being rare, emergency presentation with sudden severe, life-threatening hemorrhagic shock duodenal GISTs might be a cause of potentially lethal massive combined upper and lower gastrointestinal bleeding which is the key feature of this rare and challenging tumor.

## Introduction

Gastrointestinal stromal tumors (GISTs) are the most common mesenchymal tumors of the gastrointestinal tract, accounting for 1–3% of all gastrointestinal malignancies [1]. Duodenal stromal tumors are rare tumors among GISTs, accounting for 4–5% of GISTs [2]. Most of them develop in people over 40 years old, and there is no obvious sex difference [3]. It is primarily located in the descending and horizontal parts of the duodenum, adjacent to the liver, pancreas, and other important organs, so it has special characteristics for diagnosis and treatment. Duodenal stromal tumors are usually asymptomatic in their early stages and found incidentally. As the tumor continues to grow, there may be abdominal pain, blood in the stool, abdominal mass, intestinal obstruction, and acute peritonitis caused by perforation [4].

Surgery remains the preferred and effective treatment for GISTs. It is usually necessary to comprehensively consider the location, volume, and nature of the tumor, and the extent of invasion of surrounding tissues. The surgical principle of duodenal GISTs should be complete tumor resection to avoid tumor rupture and implantation metastasis [5]. For duodenal GISTs in special locations requiring combined organ resection, if it is difficult to perform R0 resection, imatinib can be used for preoperative treatment [6].

Due to the relative rarity of duodenal GISTs, clinicians have little understanding about its clinical features, diagnosis, treatment and prognosis. In this study, we report a case of duodenal GIST.

## Case report

A 61-year-old Egyptian man presented shocked with severe attack of hematemesis and melena, blood transfusion, resuscitation were done.

Physical examination found abdominal bulge, mass in the right upper abdomen and abdominal pain.

**Fig. 1:**
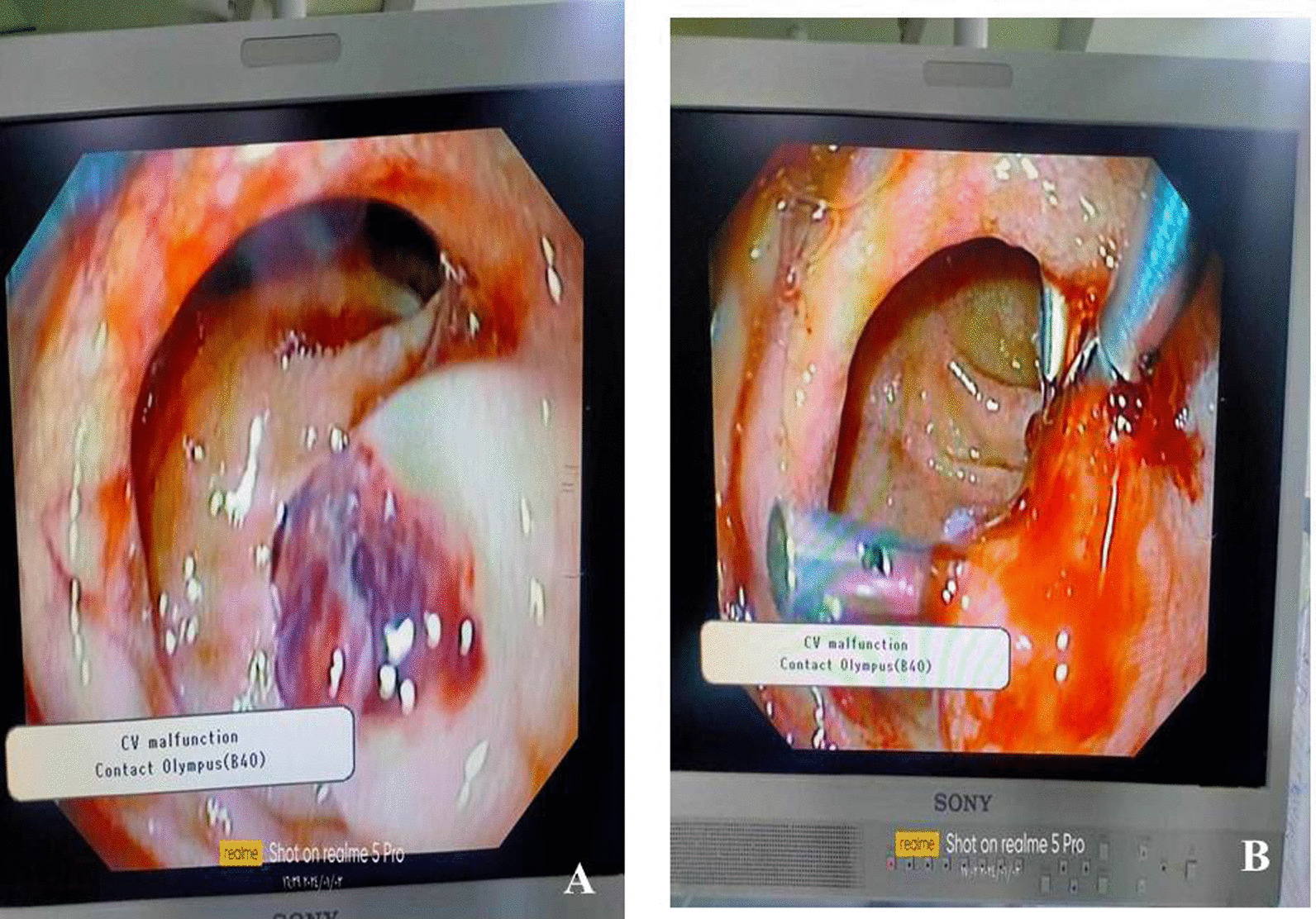
Endoscopic description of the mass: **A** Submucosal Lesion protruding into duodenal lumen with mucosal ulceration. **B** application of 4 hemoclips on the ulcerated area

Upper gastroduodenal endoscopy was done and revealed a large ulcerating bleeding mass at first part of duodenum 4 hemo-clips were applied with good homeostasis Fig. 1.

Abdominal CT scan suggested a soft tissue tumor with no peritoneal ascites in the right upper abdomen. After admission the patient underwent contrast-enhanced CT scan of the abdomen, and the scan image revealed a soft tissue mass (10.06 cm × 7.08 cm) with areas of necrosis in the right upper abdomen, suggesting a stromal tumor.

The mass appeared to be in first part of the duodenum.

**Fig. 2:**
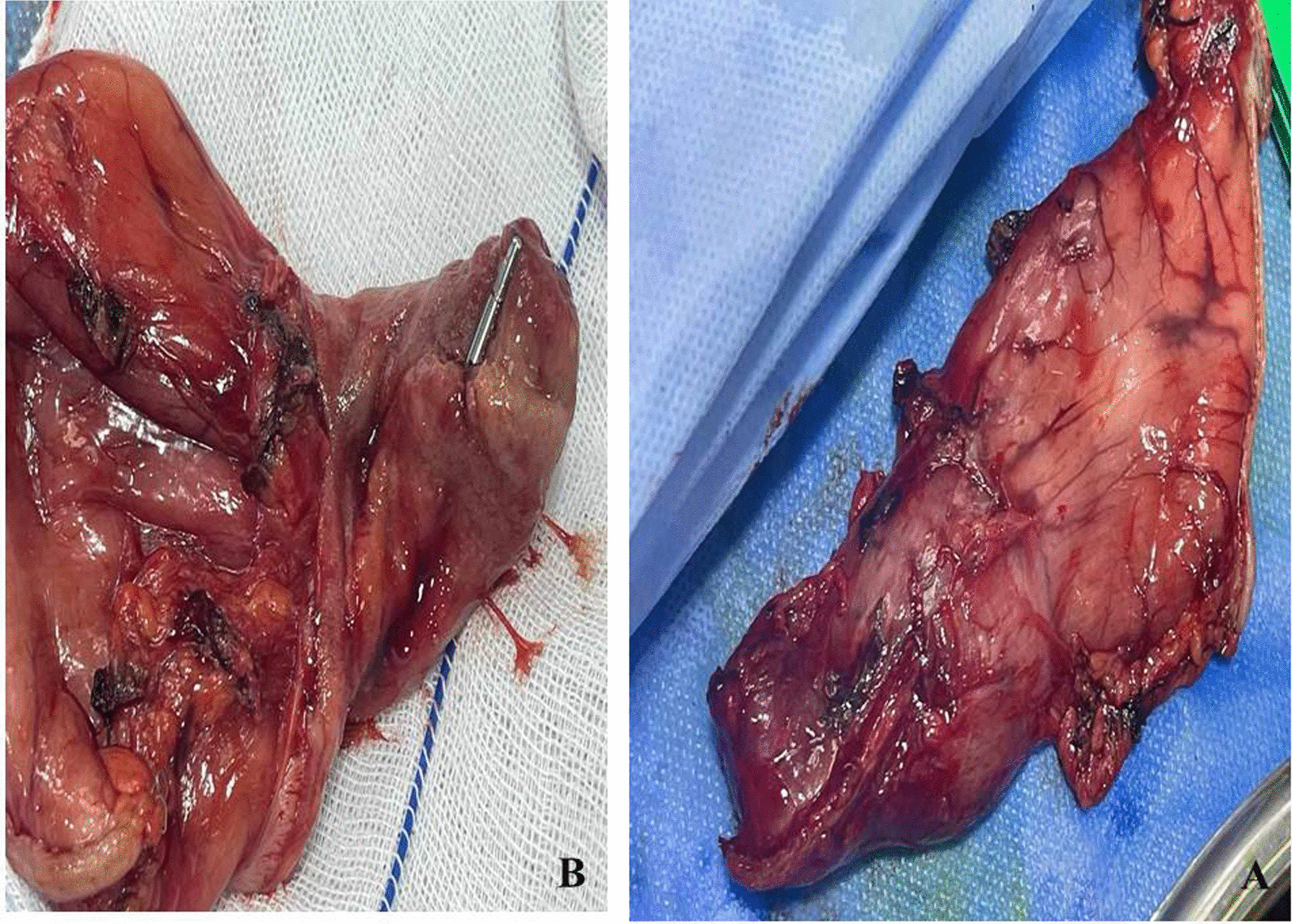
Post-operative and gross description of the mass: **A** surgically excised tissues (distal gastrectomy and duodenectomy). **B** gross description of the mass well circumscribed submucosal mass about 11 × 8× 7 cm in first part of duodenum

An exploratory laparotomy and distal gastrectomy, duodenectomy and gastrojejunostomy were performed Fig. 2.

Intra-operatively, the mass (11.0 cm × 8.0 cm) was located in first part of duodenum, with a brittle and hard texture. The surface of the mass was ulcerated and bleeds into the lumen.

**Fig. 3:**
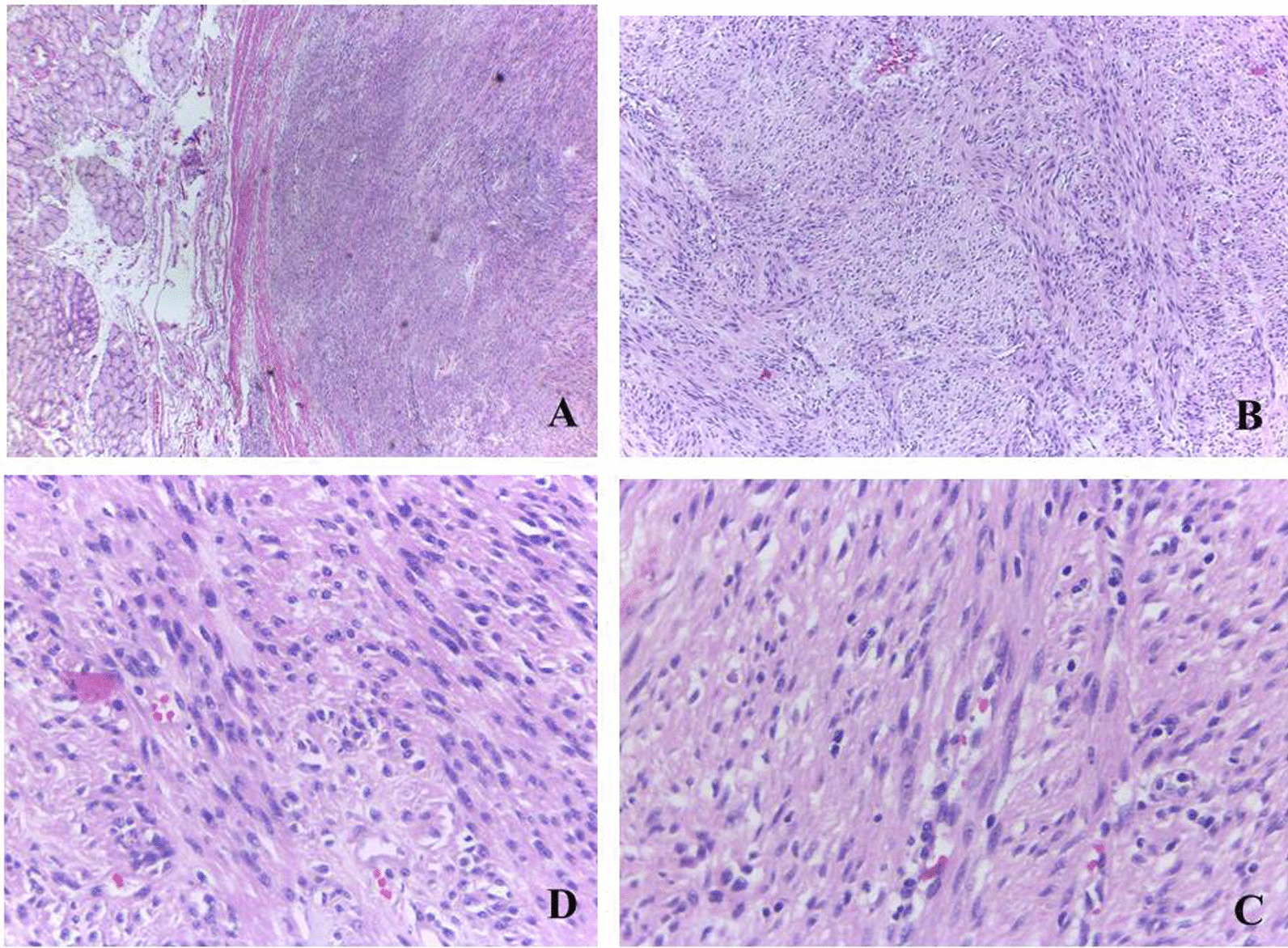
Microscopic description of the mass. **A** submucosal well circumscribed tumor tissue formed of sheets and groups of bland spindle cells. **B** high power of the tumor tissues showed fascicles and groups of spindle cells with mild to moderate nuclear atypia in a pattern less maner. **C**, **D** the tumor tissue showed mild to moderate nuclear atypia with few scatterd abnormal mitotic figures

**Fig. 4:**
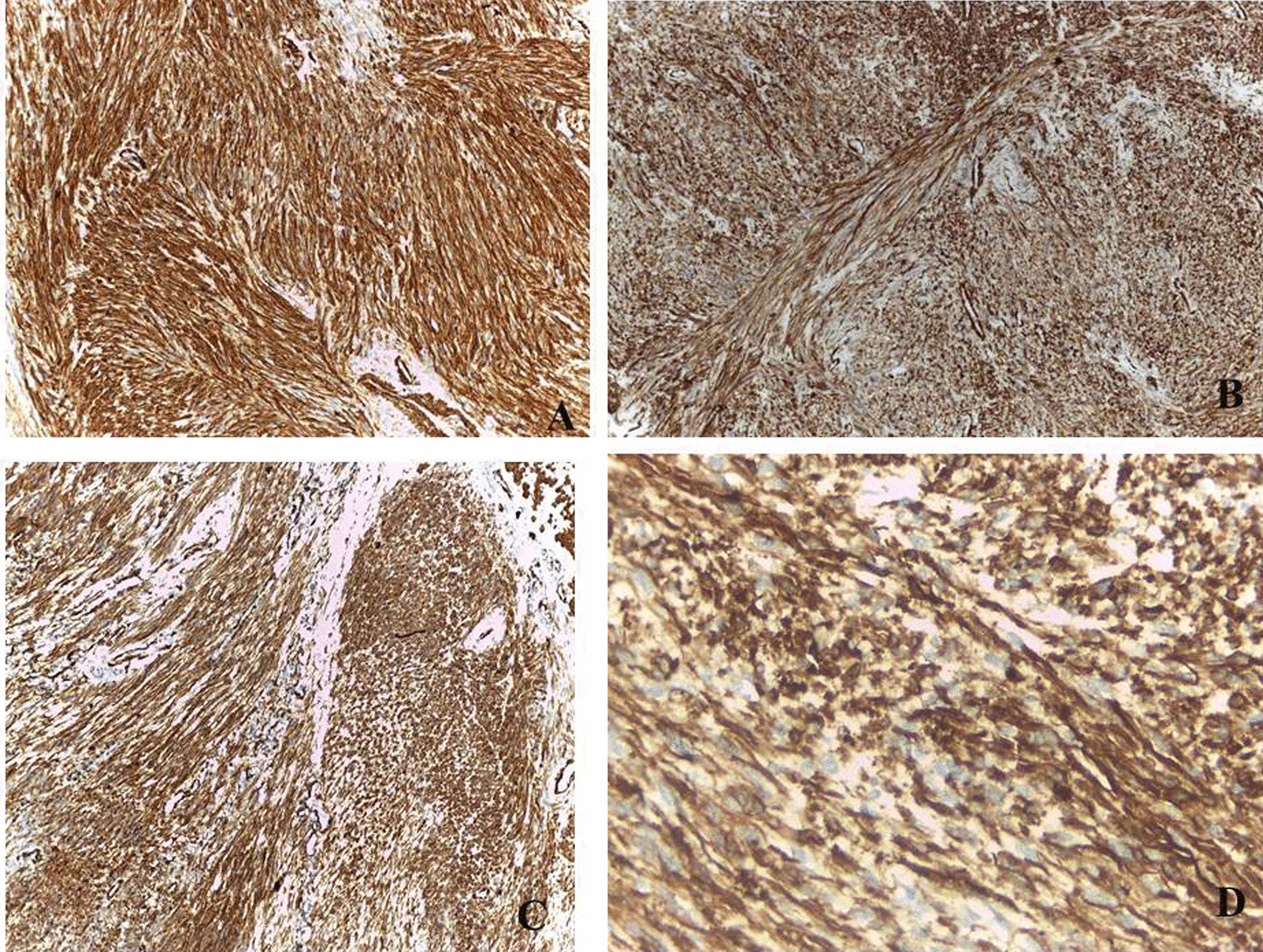
Immunohistochemical profile of the mass. **A**, **B** tumor cells showed diffuse strong positive CD117 stain. **C** tumor cells showed diffuse strong positive DOG-1 stain. **D** tumor cells showed diffuse strong positive SDHB stain

In postoperative pathology, the tumor cell morphology is shown in Fig. 3. Im-unohistochemical results (Fig. 4) were as follows, CD34 (−), desmin (−), S-100 (−), CD117 ( +), DOG-1 ( +), SDHB ( +). The morphology of the mass combined with immunohistochemistry was consistent with duodenal GIST of high risk type. Postoperatively, he recovered well without early significant complications and the hemoglobin gradually increased. The patient was discharged 10 days after the operation, and 4 months after the operation, the patient is administering amatinib one tablet daily and he was well with no evidence of tumor recurrence.

Written informed consent was obtained from the patient for publication of this case report and any accompanying images. A copy of the written consent is available for review by the Editor-in-Chief of this journal.

## Discussion

GISTs can involve the entire gastrointestinal tract, of which the stomach accounts for 60%, small intestine 25%, rectum 5%, and colon, esophagus, omentum, mesentery, and retroperitoneum less than 5% [7, 8].

Computed tomography (CT) is still the first routine examination for GISTs. It can be used not only for diagnosis and differential diagnosis, but also for evaluation of efficacy of targeted drugs and detection of tumor recurrence and metastasis [7].

The final diagnosis relies on pathology, immunohistochemistry and molecular testing.

The treatment of GIST mainly relies on surgery and molecular-targeted drugs.

GISTs have the ability for malignant transfor-mation, and the risk is closely related to tumor size, location, and mitotic figures [9].

Hu et al. [2] showed that duodenal GIST was more common in people over 50 years old. The patients were aged between 21 and 84 years, with 7 women and 13 men. Tumor size varied from 1.6 to 15 cm, with an average of 6.2 cm. The majority of tumors were located in the second part of the duodenum, and a small number of patients had tumor located in the first, third and fourth parts.

The manifestation of symptoms is mainly related to growth site, size, and relationship to the gastrointestinal wall, and whether the tumor is ruptured [10]. As the tumor grows, a pseudocapsule can be formed by compression of normal tissue, and necrosis or spontaneous rupture of the tumor can occur to a certain extent, leading to gastrointestinal perforation, hemorrhage and peritonitis [11]. Common symptoms are gastrointestinal bleeding, abdominal pain, melena, and abdominal mass. Early cases can be asymptomatic. [12, 13]. Five cases with anemia were reported [11]. Six patients (five cases from the literature plus our case) developed abdominal pain [12, 14].

Because of the atypical clinical manifestations of duodenal GIST, multiple examinations should be combined for diagnosis. GIST diagnosis mainly relies on CT, MRI, PET-CT, ultrasound endoscopy, and other related examinations [15, 16]**.**

When the grade of malignancy is high, the tumor has unclear boundaries, and can be adherent to adjacent organs, frequently with central necrosis, bleeding, cystic degeneration, and rare calcification [16–18].

The final diagnosis was performed after surgical resection by combination of morphological and immunohistochemical evaluation.

CD117 and DoG-1 are important immunohistochemical markers for the diagnosis of GIST [2].

Currently, surgery is the treatment of choice for GIST, and the main surgical options are minimally invasive endoscopic surgery, minimally invasive laparoscopic surgery and traditional open surgery. Surgical resection is considered the main method to treat duodenal GIST. Pancreaticoduodenec-tomy and local resection [15–18] were performed in all reported cases. The surgical method is chosen mainly based on the size and location of the GIST. National Comprehensive Cancer Network Clinical Practice Guidelines for GISTs (Version 2. 2022) [16] suggested that surgical resection should be performed on tumors, with negative histological margins. In the present case as the tumor was resectable and located in first part of duodenum An exploratory laparotomy and distal gastrectomy, duodenectomy and gastrojejunostomy were performed.

The patient was well without tumor recurrence, and follow-up is ongoing with administration of Imatinib daily.

For a GIST that cannot be resected or has metastases, targeted drug therapy is the main approach. Imatinib, sunitinib and regorafenib are first-, second- and third-line therapies for patients with GIST, respectively [18].

Malignant GISTs have a high recurrence and metastasis rate and an extremely poor prognosis. The 5-year survival rate after surgery for gastrointestinal mesenchymal tumors was found to be 30.5–73.2% [2].

## Conclusion

As screening equipment develops rapidly, the positive diagnosis rate of GISTs has increased. Because of its aggressiveness, early detection and treatment are of value for patients.

Our report highlights that duodenal GISTs can be presented by emergent presentation as bleeding or being asymptomatic incidentally found during abdomino-pelvic imaging tests. Final diagnosis depends on morphology and IHC.

Post-operative Imatinib intake has a good impact on the patients' survival.

## Data Availability

Available.
